# Spinal cord protection in open and endovascular aortic surgery: current strategies, controversies and future directions

**DOI:** 10.3389/fcvm.2025.1671350

**Published:** 2025-09-29

**Authors:** Debora Emanuela Torre, Carmelo Pirri

**Affiliations:** ^1^ Department of Cardiac Anesthesia and Intensive Care Unit, Cardiac Surgery, Ospedale dell’Angelo, Venice, Italy; ^2^ Department of Neurosciences, Institute of Human Anatomy, University of Padova, Padua, Italy

**Keywords:** spinal cord injury, thoracoabdominal aortic repair, thoracic aortic repair, openaortic surgery, endovascular aneurysm repair, spinal cord protection, neuroprotectionstrategies

## Abstract

Spinal cord injury (SCI) remains one of the most devastating complications following open and endovascular thoracoabdominal aortic repair, significantly affecting both survival and quality of life. Despite considerable progress in surgical and endovascular techniques, effective perioperative strategies for SCI prevention continue to evolve. This review provides a comprehensive and critical appraisal of current knowledge on the pathophysiology of spinal cord injury, along with established and emerging protective interventions. Techniques such as cerebrospinal fluid drainage, distal aortic perfusion, staged repair, permissive hypertension and intraoperative neuromonitoring are examined in light of the latest clinical evidence. The role of anatomical and hemodynamic factors, as well as pharmacological adjuncts and experimental approaches, is discussed in detail. Persistent knowledge gaps and areas of controversy, particularly regarding the optimal timing and combination of protective strategies, are identified. Future perspectives are also outlined, emphasizing the need for personalized, physiology guided approaches based on patient specific risk profiles.

## 1 Introduction

Spinal cord ischemia (SCI) remains a devasting complication of aortic interventions, profoundly affecting survival, functional outcomes and healthcare utilization (1–3). It predominantly results from hypoperfusion in spinal watershed regions, exacerbated by ischemia-reperfusion injury in open repair and by extensive segmental artery coverage in TEVAR (4, 5). Additional mechanisms include embolism and venous congestion. Established risk factors encompass patient comorbidities, procedural acuity and disruption of collateral circulation.

The spinal cord's vascular supply is inherently tenuous, relying on a dynamic equilibrium between anterior/posterior circulations and an anastomotic collateral network. Disruption of critical segmental arteries, particularly the artery of Adamkiewicz, compromises perfusion pressure gradients, precipitating infarction in metabolically vulnerable segments. Secondary injury is amplified by glutamate excitotoxicity, oxidative stress and disruption of the blood-spinal cord barrier. Contemporary mitigation strategies emphasize hemodynamic optimization, cerebrospinal fluid (CSF) drainage and augmented collateral perfusion. Pharmacologic neuroprotection (e.g., steroids, antioxidants) remains investigational, while neuromonitoring permits real-time ischemia detection (6). The primary aim of this review is to synthesize the current evidence on the pathophysiological mechanisms underlying spinal cord ischemia in thoracic and thoracoabdominal aortic surgery, encompassing both open and endovascular approaches. Particular attention is given to hemodynamic, inflammatory and microvascular disruptions that contribute to SCI, with the ultimate goal of outlining evidence-based strategies to minimize this devasting complication. A comprehensive of PubMed and Scopus was conducted from 1970 to 2025 using the terms: “spinal cord injury”, “thoracoabdominal aortic aneurysm”, “neuroprotection”, “thoracic aortic repair” and “endovascular aneurysm repair”. Additional references were retrieved through manual review of bibliographies and recent international guidelines. Experimental and clinical studies were considered, including randomized controlled trials, systematic reviews, observational cohorts and expert consensus statements; case reports and small series were included only when addressing innovative or emerging techniques. The level of evidence was explicitly graded and highlighted throughout the manuscript.

## 2 Spinal cord injury in thoracic and thoracoabdominal aortic repair: balancing open and endovascular approaches

Spinal cord injury (SCI) remains a dreaded complication of thoracic and thoracoabdominal aortic repair, with incidence rates varying significantly between two approaches. In open surgical repair, particularly Crawford extent I and II thoracoabdominal aneurysm, the incidence of permanent SCI ranges from 4% to 15%, despite contemporary neuroprotective strategies. In contrast, thoracic endovascular aortic repair (TEVAR) carries a comparatively lower, yet clinically significant, SCI risk, generally reported between 2% and 8%, depending on the length of aortic coverage, involvement of critical intercostal arteries and presence of prior infrarenal or iliac repairs. However, the relative safety of TEVAR in counterbalanced by potential limitations in managing complex anatomy or extensive disease (7–10).

Regardless of the approach, preoperative identification of SCI risk and adoption of tailored protective strategies remain paramount to improving neurological outcomes (11).

### 2.1 Spinal cord anatomy in the context of spinal cord injury

**Figure 1:**
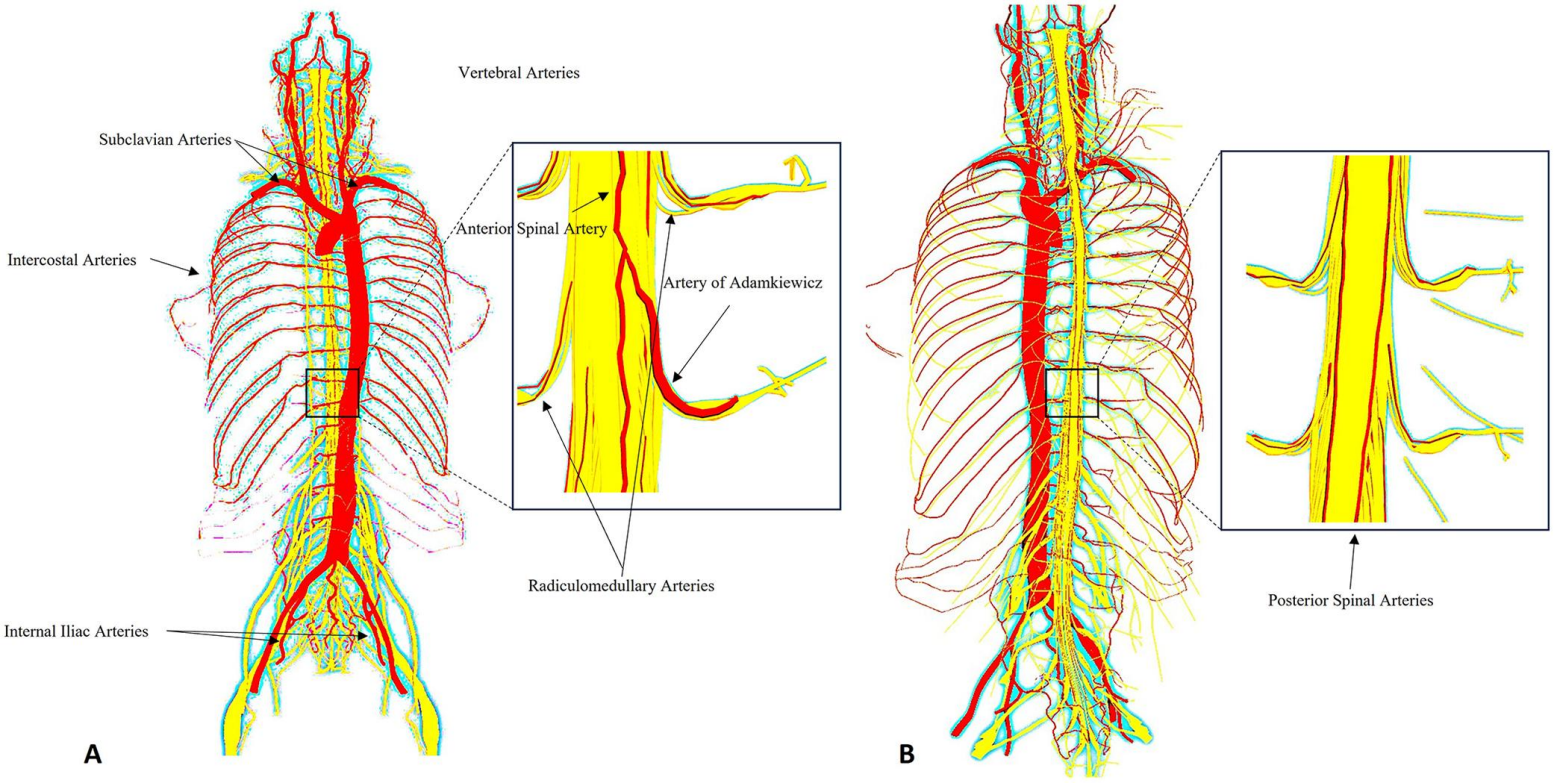
Schematic representation of the spinal cord vascular anatomy in relation to thoracic and thoracoabdominal aortic aneurysm repair. The anterior spinal artery (ASA) and paired posterior spinal arteries receive input from segmental medullary arteries, most notably the artery of Adamkiewicz (typically arising between T8 and L2). These vessels form a delicate collateral network highly vulnerable to ischemic insult during open or endovascular repair of thoracic and thoracoabdominal aortic aneurysm. The figure highlights key anatomical landmarks and vascular territories relevant to spinal cord protection strategies. **(A)** Anterior spinal arterial supply highlighting the artery of Adamkiewicz and radiculomedullary feeders. **(B)** Posterior spinal arterial network. Insets magnify the thoracolumbar segment.

The spinal cord is a cylindrical structure of central nervous tissue extending from the foramen magnum to the conus medullaris at approximately the L1–L2 vertebral level. It is encased within the vertebral canal and surrounded by cerebrospinal fluid (CSF), which provides mechanical and metabolic protection. Functionally, the spinal cord is highly organized, with gray matter (containing neuronal cell bodies) centrally located and white matter (myelinated axonal tracts) peripherally arranged. The anterior two-thirds of the spinal cord, encompassing the anterior horns and corticospinal tracts, are supplied primarily by the anterior spinal artery (ASA), while the posterior third, including the dorsal columns, receives perfusion from the paired posterior spinal arteries. The ASA arises from the vertebral arteries and receives crucial reinforcement from radiculomedullary arteries along its course. Of these, the most significant is the artery of Adamkiewicz (also known as the great anterior radiculomedullary artery), which typically arises between T8 and L2, most often on the left side. This artery plays a pivotal role in perfusing the thoracolumbar spinal cord and is particularly vulnerable during aortic interventions involving the descending thoracic or thoracoabdominal segments. Collateral flow to the spinal cord is also provided by the intercostal, lumbar and sacral segmental arteries, forming a complex longitudinal and transverse vascular network (12, 13) (Figure 1).

### 2.2 The collateral network concept

Over the past decades, the concept of the collateral network has reshaped the understanding of spinal cord blood supply. Instead of depending solely on segmental arteries (such as intercostal or lumbar arteries), the cord is perfused by an extensive system of small vessels within the spinal canal, perivertebral tissues and paraspinal muscles, interconnected with intrinsic nutrient arteries. This network receives inflow not only from intercostal and lumbar arteries but also from proximal vessels (e.g., subclavian artery) and distal contributors (e.g., hypogastric circulation), providing redundancy when a pathway is compromised. However, this adaptability entails a risk of vascular steal: during aortic cross-clamping, retrograde flow from excluded intercostal arteries (back bleeding) can divert blood away from the cord, a phenomenon exacerbated by pharmacologic vasodilation. Clinically, preserving inflow sources such as the left subclavian and hypogastric arteries and minimizing steal through careful intraoperative management, are now recognized as key strategies for preventing paraplegia (14).

## 3 Pathophysiology of spinal cord injury in aortic surgery

Spinal cord injury (SCI) after thoracic and thoracoabdominal aortic surgery is the result of a complex interplay between vascular anatomy, hemodynamics and secondary cellular events. Current knowledge emphasizes that two fundamentally distinct mechanism must be considered: ischemia-reperfusion injury (which predominates in open surgical repair following aortic cross-clamping) and sustained ischemic hypoperfusion (which is characteristic of endovascular repair). Furthermore, embolic phenomena and venous congestion represent additional contributory factors.

### 3.1 Open surgical repair: ischemia-reperfusion injury

During open thoracoabdominal aortic aneurysm repair, prolonged aortic cross-clamping interrupts the inflow through segmental intercostal and lumbar arteries, including those that provide critical supply to the anterior spinal artery. Among these, the artery of Adamkiewicz (typically arising between T8 and L2) is the principal radiculomedullary feeder to the thoracolumbar cord. Its exclusion or sacrifice is strongly associated with postoperative SCI (15). The initial ischemic insult is aggravated during the reperfusion phase, once the aorta is unclamped or intercostal arteries are reimplanted (16). Reperfusion induces a burst of reactive oxygen species (ROS) and overwhelms antioxidant defenses, causing oxidative damage. At the cellular level, reperfusion is accompanied by glutamate-mediated excitotoxicity, calcium influx and mitochondrial dysfunction. These molecular events promote disruption of the blood spinal cord barrier, infiltration of inflammatory cells and pro-apoptotic microenvironment, resulting in progressive neuronal and glial cell death (17).

Histopathological studies in animal models demonstrate selective anterior horn necrosis, consistent with the high metabolic demand and vulnerability of motor neurons (18–21).

The biphasic pattern, ischemia followed by reperfusion-mediated injury, explains why neurological deficit may be delayed after open repair, manifesting hours after surgery despite transient restoration of perfusion. This vulnerability may be further exacerbate by concomitant factors such as hemodynamic instability, anemia, sepsis or hypoxemia (22).

### 3.2 Endovascular repair: ischemic hypoperfusion

In thoracic endovascular aortic repair, the mechanism is distinct. The procedure does not involve cross- clamping and immediate reperfusion injury, but instead results in sustained hypoperfusion due to coverage and exclusion of multiple segmental arteries (23). This process reduces blood flow to the ASA and its collaterals, including the artery of Adamkiewicz and other radiculomedullary arteries (24). When collateral networks (paravertebral, iliac, subclavian, pelvic) fail to remodel adequately, spinal cord perfusion pressure may drop below the ischemic threshold, further compromised by a reduction in mean arterial pressure (MAP) or an increase in cerebrospinal fluid pressure (CSFP), for example due to edema or impaired drainage (25). The risk is greatest in the mid-thoracic watershed zone (T4–T8), where intrinsic collateral density is sparse (26, 27).

Animal ligation models mimicking segmental artery exclusion demonstrate immediate paralysis when perfusion capacity falls below a critical threshold. Unlike reperfusion injury, which is oxidative and inflammatory in nature, hypoperfusion injury is characterized by progressive energy failure and neuronal death due to insufficient blood flow (28–30).

### 3.3 Embolic phenomena and venous congestion

Although ischemia-reperfusion and ischemic hypoperfusion represent the principal mechanism underlying SCI, embolic events constitute an additional but less common contributory pathway that may occur in both open and TEVAR repair. In open surgery, extensive aortic manipulation, clamping and unclamping, as well as retrograde flushing can dislodge mural thrombus or atheromatous debris. In TEVAR, emboli may also consist of thrombus, atheromatous debris, air, or even foreign material from guidewires, sheaths or stent graft coatings (30–32). Their impact is particularly deleterious when they obstruct radiculomedullary arteries such as the Adamkiewicz or other major feeders.

Embolic occlusion produces a localized perfusion deficit in the territory of the obstructed vessel. Due to its high metabolic demand and poor collateral reserve, the anterior gray matter is particularly vulnerable and therefore reaches the ischemic threshold earlier than surrounding tissue, resulting in focal infarction. The spinal cord microcirculation further contributes to this vulnerability, as its terminal branches such as sulcal arteries, have limited collateral redundancy and a reduced capacity to clear embolic material, often leading to delayed or absent reperfusion (33, 34).

This can result in patchy or asymmetric neurologic deficits distinct from those due to global hypoperfusion. Moreover, the spinal microcirculation lacks a robust capacity for embolic clearance and reperfusion is often delayed or absent due to the limited collateral redundancy of these terminal branches (30).

Another contributory mechanism is venous congestion. During extensive TEVAR, exclusion of intercostal arteries compromises segmental arterial inflow and predisposes to spinal cord ischemia. Once ischemic injury has developed, intramedullary edema and impaired microcirculatory drainage raise venous pressure within the cord, thereby reducing the arteriovenous gradient required for adequate perfusion and further amplifying neurological damage.

In open surgical repair, aortic cross-clamping acutely elevates central venous pressure and cerebrospinal fluid pressures, further lowering SCPP and worsening ischemia.

Spinal venous pressure may also be secondarily increased by systemic factor such as mechanical ventilation with high levels of positive end expiratory pressures (PEEP) or right heart dysfunction, further worsening perfusion compromise (35).

## 4 SCI classification and risk factors

SCI may present as paraplegia (complete motor loss) or paraparesis (partial preservation), with onset either immediate (≤24 h) or delayed (>24 h) (36). Open surgical repair may cause immediate paralysis from ischemia due to cross clamping and segmental artery sacrifice, often compounded by reperfusion injury. Delayed SCI usually arises from secondary insults such as hypotension, anemia or arrhythmias. TEVAR can also lead to SCI, which may be delayed, as progressive failure of collateral perfusion follows extensive segmental artery coverage and endoleak resolution, or early, when large aortic segments are covered without adequate collateral reserve, causing abrupt hypoperfusion of critical spinal cord territories (16, 30, 31, 37). Transient forms resolve within 30 days, while deficits persisting beyond this interval are considered permanent (36).

Understanding the risk factors associated with SCI following both open and endovascular repair of thoracoabdominal and thoracic aortic aneurysm is crucial for refining patient selection and optimizing perioperative strategies.

Despite differences in the technical approaches, several risk factors are consistently implicated across both modalities, reflecting the fundamental vulnerability of the spinal's cord perfusion architecture.

**Figure 2:**
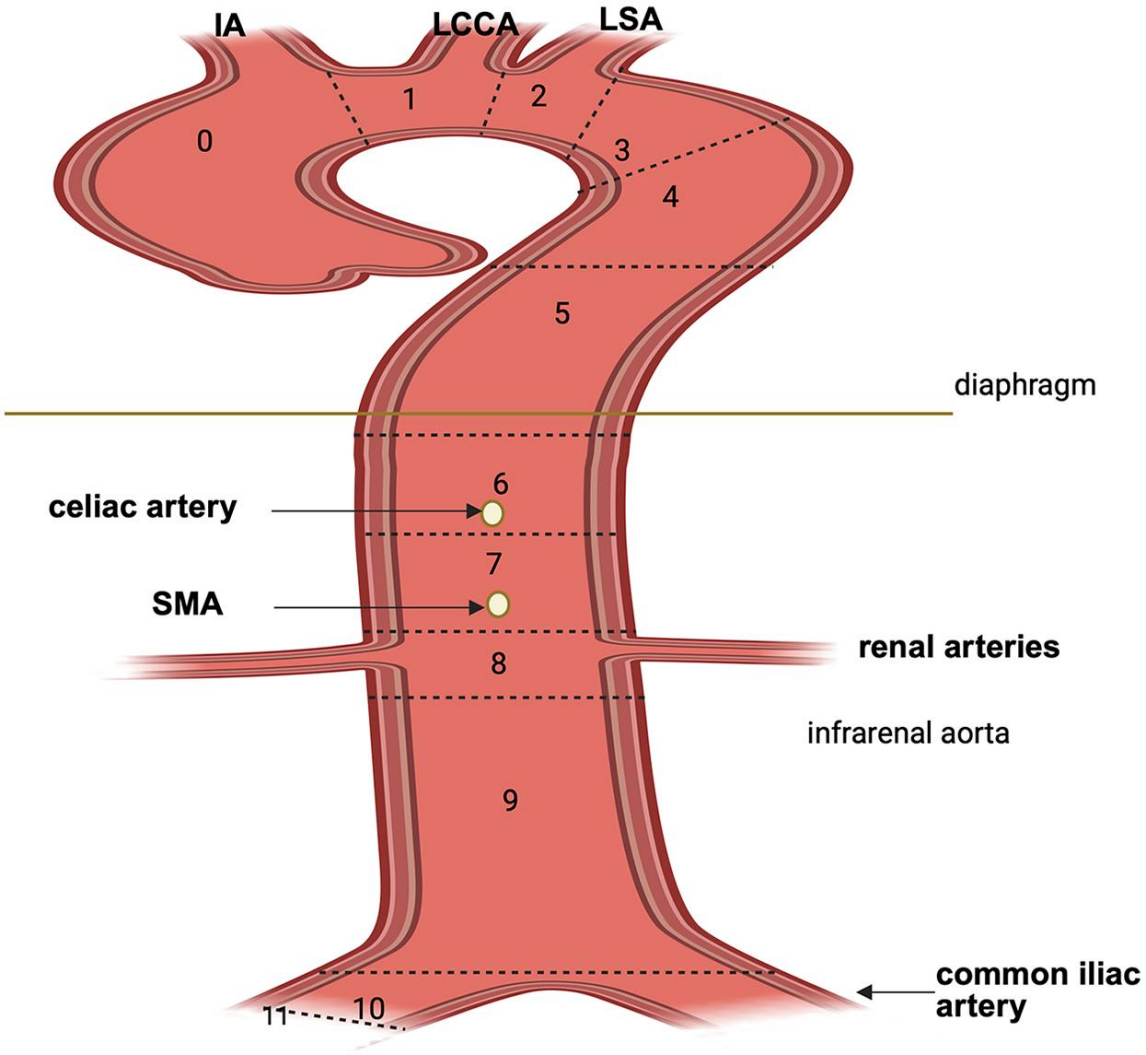
Aortic arch zone classification according to Society for Vascular Surgery (SVS). Zone 0: comprises the ascending aorta and the origin of the innominate artery; zone 1: includes the origin of LCCA; zone 2: includes the origin of the LSA; zone 3: extends from the distal aspects of the LSA and comprises the first 2 cm of the descending aorta; zone 4: extends from the end of the zone 3 through the descending aorta (T6); zone 5: mid descending aorta to celiac artery; zone 6: celiac artery to SMA; zone 7: SMA to renal arteries; zone 8: renal to infrarenal abdominal aorta; zone 9: infrarenal aorta; zone 10: common iliac arteries; zone 11: external iliac arteries. IA, innominate artery; LCCA, left common carotid artery; LSA, left subclavian artery; SMA, superior mesenteric artery. Created in BioRender. Pirri, C. (2025) https://BioRender.com/1zh1pwi, licensed under Academic License.

The extent of aortic coverage or replacement is the most consistently determinant of SCI risk. In open repair, risk scales with aortic clamp level and interruption of critical intercostals, particularly T8-L1. In endovascular repair, longer thoracic coverage correlates with higher SCI incidence, with a 30% increased risk per additional 20 mm of coverage; distal landing in Ishimaru zones 5–10 is a specific intraoperative risk factor because it typically requires longer thoracic coverage and encroach on the thoracoabdominal junction, sacrificing more intercostal and lumbar segmental arteries within T8-L1 corridor, including the potential origin of the artery of Adamkiewicz (Figure 2).

Extensive aortic manipulation, especially in Crawford type II aneurysm, carries the highest neurologic risk in both settings (38, 39).

Procedure duration and technical complexity are also significant contributors. Prolonged cross-clamp time in open surgery aggravates ischemic insult (35, 40). In TEVAR, extended fluoroscopy times and high contrast volumes associate with increased SCI risk, likely reflecting more extensive or technically challenging interventions (41); longer procedural time may also increase embolic burden and prolong spinal cord hypoperfusion.

Perioperative hemodynamic instability, particularly sustained intra or postoperative hypotension, is a critical and potentially modifiable risk factor in both techniques. As spinal cord perfusion pressure depends on the difference between mean arterial pressure (MAP) and cerebrospinal fluid pressure, (CSFP), inadequate MAP, especially in the presence of elevated CSFP or impaired autoregulation, can critically reduce spinal cord blood flow. Episodes of hypotension in the early postoperative period are particularly associated with delayed onset of SCI (42).

Pre-existing vascular comorbidities, such as peripheral arterial disease (PAD) and chronic kidney disease (notably baseline eGFR < 30 ml/min), independently increase SCI risk by compromising collateral network integrity and perfusion reserve (43, 44). Systemic hypertension may predispose to SCI by shifting autoregulatory thresholds, requiring higher perfusion pressures to maintain spinal cord oxygenation (45).

Prior aortic interventions increase risk by cumulatively reducing collateral pathways through prior coverage or surgical exclusion (46).

Urgent or emergent interventions (e.g., rupture, acute aortic syndromes) carry additional risk because optimal preoperative planning is limited, hemodynamics are often compromised and protective strategies cannot be fully implemented (47).

### 4.1 Risk stratification models for SCI

Identifying predictive risk factors for SCI is crucial for developing preoperative stratification models and guiding prophylactic cerebrospinal fluid drainage. However, only a few scoring systems have been validated for clinical use. In a large retrospective study of 7,889 TEVAR patients, Mousa et al. (48) developed a prediction model incorporating 13 demographic and procedural variables, enabling risk stratification into defined categories and supporting clinical decision-making for spinal cord protection (Table 1).

**Table 1:** Risk scoring for SCI adapted from Mousa (48).

Variable	Score
Age (by decade)	0.5
Celiac coverage	1
Current smoker	1
Dialysis	1.5
≥3 aortic devices	1
Emergent/urgent surgery	1
Adjunct aortic procedure	1.5
Adjunct non-aortic procedure	1.5
Device length 19–31 cm	1.5
Device length ≥32 cm	3
ASA class 4–5	1
Procedure time ≥ 154 min	1
High volume center (≥50)	−1
eGFR ≥ 60 ml/min	−1
**Low risk**	**Score 0–4**
**Medium risk**	**Score 4.5–6–5**
**High risk**	**Score ≥7**

ASA, American society of anesthesiologist; CKD, chronic kidney disease; eGFR, estimated glomerular filtration rate.

### 4.2 SCI in aortic dissection

Across the aortic dissection spectrum, SCI is uncommon but clinically devasting.

Urgent or emergent presentations increase the risk because unstable physiology, limited preoperative planning and constrained implementation of protective measures amplify vulnerability (47). As an initial presentation, SCI is reported in a minority of acute dissection. Clinical presentation vary according to the vascular territory involved, with neurological severity ranging from complete paraplegia to mild weakness. Back pain is common, reported in up to 70% of cases, and typically localizes to the level of the lesion (49–51).

After repair, incidence depends on approach and extent of aortic coverage. In open/arch repair and frozen-elephant trunk (FET) for type A dissection, contemporary systematic reviews report SCI 0.8%–8.9%, with center experience, distal extension of the stented segment, hypotension and prior aortic procedures modulating risk. Some series indicate that, in appropriately selected acute type A aortic dissection patients, FET does not raise paraplegia risk compared with conventional strategies (52, 53).

In TEVAR for type B dissection, pooled analyses consistently show lower SCI rates than TEVAR for degenerative aneurysm, but non negligible risk remains; reported ranges across observational cohorts are roughly 0.8%–8.6%, with longer thoracic coverage, distal landing near the celiac axis, perioperative hypotension/anemia, LSA coverage without revascularization and prior aortic surgery as key predictors (54).

### 4.3 Clinical manifestations of SCI after TAA and TAAA repair

The clinical spectrum of SCI ranges from mild motor deficits to complete paralysis, with immediate or delayed onset. Autonomic involvement may occur, causing cardiovascular, thermoregulatory and bladder and bowel dysfunction (55–57). Disruption of descending vasomotor tracts, sympathetic preganglionic neurons and spinal afferents impairs autonomic vascular control and can produce hypotension and bradycardia (spinal shock) (57). In delayed paraplegia, Cheung et al. (58) observed neurological recovery accompanied by decreasing vasopressor requirements, consistent with restoration of autonomic function. Thus, hypotension is both a contributor to spinal ischemia and a potential early marker of autonomic spinal cord involvement.

### 4.4 Secondary spinal cord injury (SSCI)

Secondary spinal cord injury, evolving over hours to days after the primary ischemic insult, comprises interrelated cellular, biochemical, vascular and inflammatory cascades that worsen tissue injury. It may be local (intraspinal metabolic/inflammatory responses to ischemia), or systemic, due to impaired cardiopulmonary function with inadequate spinal perfusion. The latter subtype, termed secondary spinal cord injury of systemic origin, explains the heightened vulnerability in patients with comorbidities such as chronic obstructive pulmonary disease (COPD) or perioperative anemia, which reduce oxygen delivery. Perioperative hypoxemia is a modifiable risk factor (59); maintaining a partial pressure of arterial oxygen (PaO_2_ ≥ 60 mmHg (corresponding to spO_2_ > 90%) and Hb levels ≥ 10 g/dl are a fundamental targets within spinal cord protection bundles (60).

## 5 Strategies to minimize SCI

**Figure 3:**
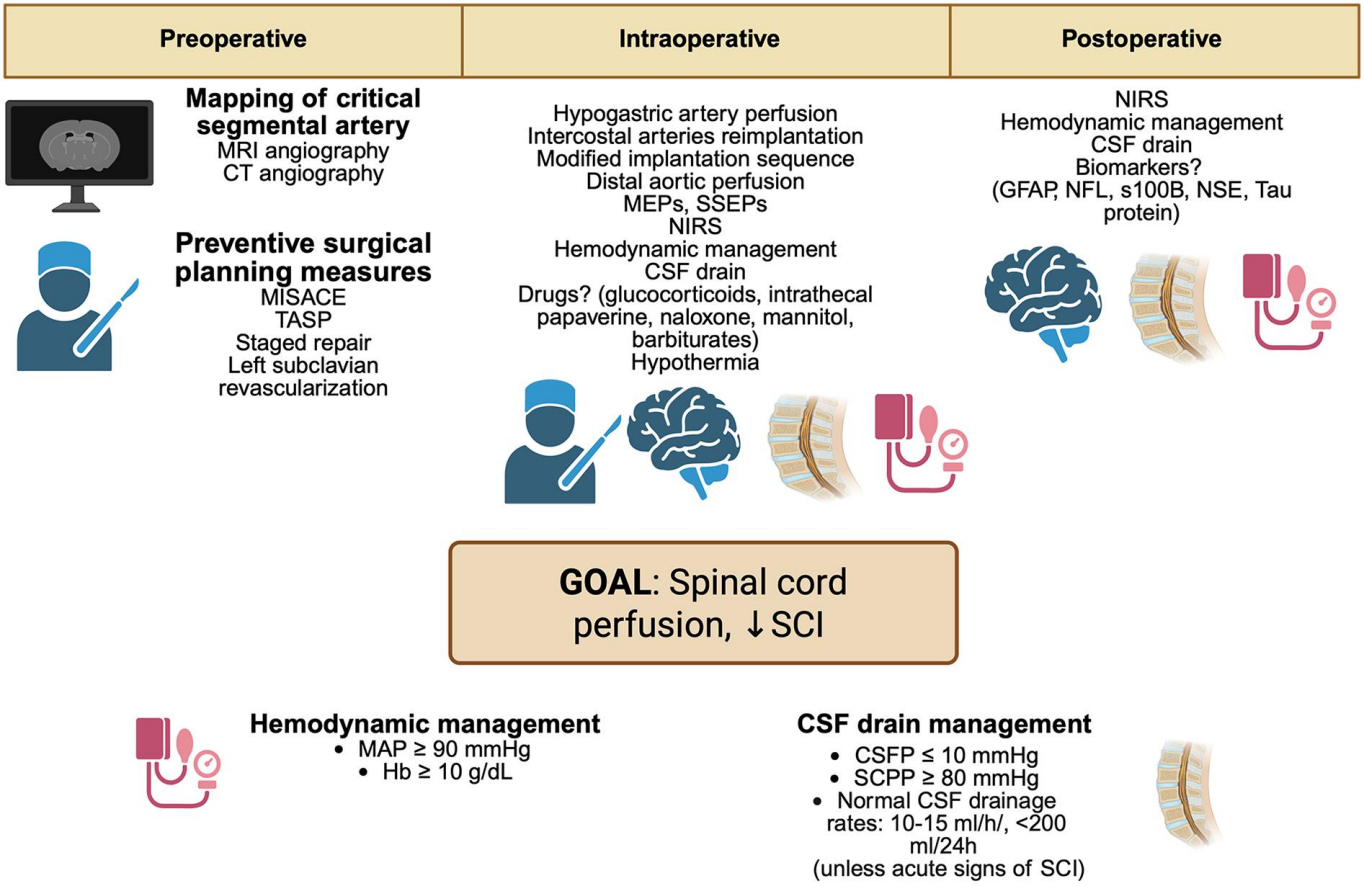
Perioperative strategies to minimize SCI in aortic surgery. MRI, magnetic resonance imaging; CT, computed angiography; MISACE, minimally invasive segmental artery coil embolization; TASP, temporary aneurysm sac perfusion; TEVAR, thoracic endovascular aortic repair; MEPs, motor evoked potentials; SSEPs, somatosensory evoked potentials; NIRS, near-infrared spectroscopy; MAP, mean arterial pressure; Hb, hemoglobin; CSF, cerebrospinal fluid; CVP, central venous pressure; CSFP, cerebrospinal fluid pressure; SCPP, spinal cord perfusion pressure; GFAP, glial fibrillary acidic protein; NFL, neurofilament light chain; S100B, S100 calcium-binding protein B; NSE, neuron-specific enolase. Created in BioRender. Pirri, C. (2025) https://BioRender.com/oh1lb9s, licensed under Academic License.

Strategies to minimize SCI in patients following TAA and TAAA repair could be recognized in these approaches: 1) Preoperative planning ed evaluation; 2) perioperative strategies (Figure 3).

### 5.1 Preoperative planning and intraoperative strategies

#### 5.1.1 Role of the left subclavian artery in SCI

The left subclavian artery (LSA) is pivotal for perfusion of the posterior cerebral circulation, upper extremity and spinal cord through contribution to the vertebral, internal thoracic and intercostal arteries, which anastomose with the anterior spinal artery and the artery of Adamkiewicz. Its integrity must therefore be carefully evaluated before thoracic and thoracoabdominal aortic repair. In TEVAR, LSA coverage (often required in cases involving aortic arch Ishimaru zones 2–3) without revascularization increases the risk of upper limb ischemia, vertebrobasilar insufficiency and spinal cord ischemia (61–63). Mitigation strategies include left common carotid artery (LCCA)-LSA transposition, LCCA-LSA bypass and thoracic branched endografts (64, 65). While some advocate routine revascularization, current evidence favors a selective approach tailored to anatomy and risk profile (66). Revascularization is mandatory in high-risk scenarios: dominant left vertebral artery (67), contralateral vertebral occlusion/hypoplasia, impaired spinal collateral flow, previous infrarenal repair with lumbar/middle sacral ligation, planned thoracic coverage >200 mm, hypogastric artery occlusion or prior left internal mammary artery (LIMA) grafting. Preoperative assessment of the circle of Willis and cerebral collaterals is crucial. The Society for Vascular Surgery (SVS) guidelines recommend revascularization in cases of dominant left vertebral artery, prior LIMA-left anterior descending (LAD) bypass or extensive aortic coverage (68), (Table 2). In open TAA/TAAA repair, especially Crawford extent II–III, the LSA remains a cornerstone of spinal cord protection, either through direct reimplantation onto the graft or via LCCA-LSA bypass (14), (Table 3). Large surgical series confirm that preserving antegrade flow through vertebral and intercostal branches reduces paraplegia risk (69). Failure to preserve or reconstruct the LSA compromises collateral perfusion and significantly increases SCI risk. Thus, in both TEVAR and open repair, LSA management must be individualized and integrated in spinal cord protection protocols.

**Table 2:** Indications for LSA revascularization based on society for vascular surgery guidelines (55).

Indications	GRADE	Quality
Extensive aortic coverage >20 cm	1	C
Termination of the LVA into PICA		
Absent/Atretic or occluded RVA		
Prior LIMA-LAD bypass		
Hypogastric artery occlusion		
Hypogastric artery occlusion		
Patent left arm AV shunt for dialysis		
Prior infrarenal aortic repair with ligated lumbar/middle sacral arteries		
Early distal thoracic aneurysm anticipating future repair		
Elective TEVAR requiring LSA coverage	2	C
Acute thoracic emergencies		

LSA, left subclavian artery; LIMA, left internal mammary artery; LAD, left anterior descending artery; TEVAR, thoracic endovascular aortic repair; PICA, posterior inferior cerebral artery; LVA, left vertebral artery; RVA, right vertebral artery; AV, arteriovenous.

**Table 3:** Management of the left subclavian artery in patients undergoing TEVAR or open TAA/TAAA repair.

Variable	TEVAR	Open TAA/TAAA repair
Timing	Preoperative (first-stage revascularization)	Intraoperative
Surgical access	Supraclavicular cervicotomy	Thoracotomy/sternotomy
Type of revascularization	Extra-anatomical (carotid-subclavian bypass or transposition)	Extra-anatomical or direct reimplantation onto the graft
Procedural context	Hybrid/minimally invasive	Open surgery with CPB or aortic clamping

TEVAR, thoracic endovascular aortic repair; TAA, thoracic aortic aneurysm; TAAA, thoracoabdominal aortic aneurysm; CPB, cardiopulmonary bypass.

Evidence appraisal: preservation or revascularization of the LSA is guideline-endorsed on the basis of observational data and expert consensus (Class IIa, LoE C).

#### 5.1.2 Role of the internal iliac (hypogastric) arteries in spinal cord perfusion

The internal iliac (hypogastric) arteries are key contributors to distal spinal cord perfusion via radiculomedullary branches originating from lumbosacral arteries (70, 71). Preserving their patency is critical in both open and endovascular aortic repair, especially when extending to the aortoiliac bifurcation (72). In endovascular aneurysm repair, unilateral embolization is often required (for endograft extension into the external iliac artery and to prevent type II endoleaks arising from retrograde perfusion via the internal iliac trunk) and is generally well tolerated thanks to pelvic collaterals, whereas bilateral occlusion markedly increases the risk of buttock claudication, erectile dysfunction and occasionally SCI (73). During TEVAR, hypogastric flow may be compromised by large-bore iliac conduits; in a cohort of 153 patients, Khoynezhad et al. (74) reported a 4.4% SCI incidence, mostly in those requiring iliac access. Preoperative imaging to confirm bilateral hypogastric patency is therefore essential. When iliac bifurcation aneurysm are present, iliac branch devices (IBDs) allow exclusion while preserving hypogastric flow. In a prospective series of 157 patients undergoing endovascular repair with IBDs, Simonte et al. (75) reported no SCI, while Schneider et al. (76) demonstrated durable outcomes with >95.1% primary patency rate of the internal iliac limb and >90.5% freedom from reintervention at 5 years, supporting the durability and efficacy of iliac branch devices in preserving pelvic and spinal cord perfusion. Thus, the hypogastric arteries are crucial to the spinal cord collateral network and preservation, either by avoiding bilateral embolization or through IBDs, should be a standard objective in aortic repair planning.

Evidence appraisal: recommendations to preserve at least one hypogastric artery rely on observational evidence and physiological rationale (LoE C).

#### 5.1.3 Reimplantation of intercostal arteries

Extensive coverage of the thoracoabdominal aorta can critically impair segmental spinal cord perfusion by compromising intercostal and lumbar arterial inflow, thereby increasing the risk of SCI (38). Afifi et al. (77), in an extensive series of 1096 open TAA and TAAA repairs, showed that ligation of intercostal arteries between T8 and T12 significantly raised the incidence of postoperative paraplegia. Notably, the intercostal artery reimplantation adds minimal operative time, yet may substantially mitigate SCI risk.

Evidence appraisal: decision on intercostal reimplantation are driven by case-by case risk-benefit assessment; evidence remains observational and consensus-based (LoE C).

#### 5.1.4 Staged strategy

In the context of extensive TAA and TAAA requiring prolonged aortic coverage, a staged repair strategy, whether open or endovascular, has emerged as a key approach to mitigate the risk of SCI. This technique involves dividing the repair into two sequential procedures, allowing time for the development and reinforcement of collateral perfusion pathways. Etz et al. (78) reported significantly lower rates of paraparesis and paraplegia in patients undergoing staged open TAAA repair, despite a greater number of sacrificed segmental arteries. Similarly, O'Callaghan et al. (79) demonstrated a reduced incidence of SCI following staged endovascular repair, with all neurologic deficits being transient, even though the staged group had longer aortic coverage. Regarding timing, there is no universally accepted interval between staged procedures and decision should be individualized based on aortic pathology, patient comorbidities and SCI risk (80).

Observational series suggest that completion after 2–8 weeks allows for collateral network maturation while minimizing interval aortic events (aortic rupture, aneurysm expansion, aortic dissection, malperfusion events) (81).

More elective programs describe intervals of up to 8–12 weeks or longer (80). Prolonged staging (>3 month) has also been reported in highly selected, stable patients under close surveillance (79).

Experimental animal models have demonstrated that staged repair can be safely completed after an interval as short as 7 days, particularly in swine studies showing preservation of collateral network perfusion (82).

Evidence appraisal: staged repair appears associated with lower SCI rates in observational registries, but randomized evidence is lacking; current guidance reflects expert consensus (LoE C).

#### 5.1.5 Minimally invasive segmental artery coil embolization (MISACE)

Minimally invasive segmental artery coil embolization (MISACE) is an emerging preconditioning technique designed to reduce the risk of SCI in patients undergoing TAA or TAAA repair. The underlying rationale is to induce adaptive neoangiogenesis by embolizing selected segmental arteries before the index procedure, thereby enhancing collateral spinal cord perfusion. Branzan et al. (83) validated this technique in a cohort of 57 TAAA patients, achieving embolization of 77.7% of targeted segmental arteries and reporting no SCI events. Addas et al. (84) emphasized the importance of meticulous preoperative imaging to identify anatomical landmarks for accurate targeting, coil sizing and procedural planning. In their series of 17 patients, all procedures were performed via transfemoral approach, with coils deployed proximally in the segmental artery and a median interval of 51.2 days between MISACE and definitive aneurysm repair. These results are confirmed by Haunschild et al. (85) in their multicentric pilot study, demonstrating the MISACE potential to reduce postoperative paraplegia. Despite promising outcomes, MISACE is not without risks. Potential complications include incomplete occlusion due to concurrent antithrombotic therapy, coil migration into the aorta, procedural complexity due to vessel tortuosity or size, high contrast load, prolonged fluoroscopy time (84). In a case report, Banks et al. (86) report recent advancements in imaging technologies that have significantly improved the precision and safety of complex endovascular procedures, such as MISACE. 3D fusion CT system and fiber optic real shape (FORS) imaging represent transformative tools that enhance intraoperative navigation while minimizing radiation exposure. 3D fusion CT overlays a preoperative CT angiogram onto live fluoroscopy, aligned with bony landmarks to provide real time multi angle anatomical guidance. FORS technology uses fiberoptic embedded guidewires to track wire position without radiation, enabling precise vessel cannulation under direct visualization.

Evidence appraisal: evidence consist of small prospective series and pilot studies without randomized comparisons; the approach remains investigational (LoE C).

#### 5.1.6 Temporary aneurysm sac perfusion (TASP)

The temporary aneurysm sac perfusion (TASP) technique is a staged endovascular approach aimed at reducing the risk of SCI during thoracic endovascular aortic repair. The initial phase involves the intentional creation of a type II endoleak by deploying a branched endograft with a side branch perfusing the aneurysm sac and a major splanchnic vessel. After a latency period of 1–3 months, a second procedure is performed to complete the visceral revascularization by bridging the branch with a stent to the target vessel, thereby fully excluding the aneurysm sac. A prospective study by Kasprzak et al. (87) involving 83 patients (40 with TASP, 43 without) demonstrated a significant reduction in the incidence of SCI in the TASP group (5% vs. 21%). Despite this promising outcome, TASP is associated with certain risks, including a higher incidence of temporary paraparesis and paresthesia (30%), the potential for persistent endoleak and the theoretical risk of aneurysm rupture during the interstage period. These findings warrant further investigation to better define the safety profile and long-term outcomes of this techniques (87, 88).

Evidence appraisal: data derive from single-center prospective but non-randomized experience; benefits are plausible but unconfirmed and the strategy should be considered investigational (LoE C).

#### 5.1.7 Implantation sequence rearrangement for branched/fenestrated stents

In standard branched or fenestrated endograft procedures, the final phase involves the deployment of the bifurcated main body and iliac limbs via large caliber sheaths introduced through the femoral or iliac arteries. These sheaths temporarily obstruct pelvic and lower limb perfusion, consequently compromising collateral spinal cord circulation. To mitigate this risk, some centers have adopted a modified implantation sequence. This revised approach entails earlier deployment of the central stent graft segment, followed by prompt withdrawal of the large sheaths to re-establish iliac and femoral perfusion earlier during the procedure. Preliminary reports suggest this strategy may lower the incidence of the spinal cord ischemia (89, 90).

Evidence appraisal: evidence for implantation-sequence rearrangement is observational/consensus (no RCTs); benefits is biologically plausible (earlier pelvic/limb flow) and supported by cohort data linking early reperfusion to lower SCI but no outcome level randomized proof exists (LoE C).

#### 5.1.8 Mapping of critical segmental arteries

Preoperative mapping of critical segmental arteries (CSA), particularly the artery of Adamkiewicz, involves the use of advanced imaging modalities such as high-resolution CT angiography or MR angiography, to identify the main arterial feeders of the spinal cord. This process aims to delineate the origin, trajectory and vertebral entry points of critical segmental arteries that contribute to spinal cord perfusion, particularly within the thoracolumbar region (typically T8 to L2). Accurate mapping enables surgical team to tailor intraoperative strategies. Specifically, it helps to identify the segmental artery supplying the Adamkiewicz artery in order to avoid inadvertent interruption during aortic cross-clamping or stent deployment, to assess the adequacy of collateral flow should a segmental vessel be sacrificed, to determine the need for segmental artery reimplantation during open repair and to integrate anatomical data with intraoperative neuromonitoring for real time decision making (91–93).

Evidence appraisal: evidence for segmental artery mapping and targeted reimplantation is limited to observational series with heterogeneous outcomes and no randomized trials. Accordingly, guideline support is weak and CSA mapping is considered investigational (LoE C).

### 5.2 Perioperative strategies

#### 5.2.1 Neuromonitoring

Intraoperative neuromonitoring (IONM) provides real-time assessment of spinal cord function during open and endovascular aortic repair (94, 95). Motor evoked potentials (MEPs) reflect anterior motor pathways, while somatosensory evoked potentials (SSEPs) assess dorsal column integrity (96, 97). MEPs are particularly sensitive to ischemia, with rapid suppression within minutes, enabling timely corrective actions (98). Persistent intraoperative MEPs loss correlates with postoperative SCI, highlighting their clinical value. Limitations include susceptibility to anesthetic effects (e.g., SSEPs attenuation by volatile agents), artifacts from limb ischemia during sheath placement and inability to distinguish moderate from severe injury (99, 100). Near-infrared spectroscopy (NIRS) offers a non-invasive adjunct by monitoring paraspinal oxygenation as a surrogate for spinal cord perfusion (101, 102). Its use extends into postoperative care, but validated thresholds are lacking, limiting prognostic utility.

Evidence appraisal: the support for intraoperative neuromonitoring is largely observational, with no RCTs showing improved neurological outcomes; recommendations are therefore consensus-based (LoE C) and remain institution-dependent.

NIRS is considered investigational owing to the lack of validated thresholds and outcome-driven evidence (LoE C).

#### 5.2.2 Blood pressure management and spinal cord perfusion pressure

Maintaining adequate spinal cord perfusion is a cornerstone of spinal cord ischemia prevention during endovascular and open aortic repair. This entails ensuring sufficient systemic oxygen delivery through maintenance of a normal cardiac index and adequate hemoglobin concentration (≥10 g/dl) (47). Notably, strategies to augment cardiac output must be carefully selected: volume expansion can increase central venous pressure (CVP) and promote tissue edema, potentially impairing spinal cord perfusion, while adrenergic inotropes may exert deleterious effects at the microvascular level of the spinal cord. Spinal cord perfusion pressure (SCPP) is determined by the gradient between mean arterial pressure (MAP) and the greater of CSFP or CVP (99, 103). While mean CSFP is commonly used as a benchmark for evaluating drainage and monitoring, this approach presents several technical challenges, including a wide physiological range, variability and susceptibility to errors related to zeroing levels. These limitations highlight the need for more advanced methods of CSFP signal analysis, which have recently emerged from the spinal cord research field. Future cross-disciplinary collaborations between spinal cord and cardiovascular monitoring experts may ultimately enable the development of personalized CSFP-derived metrics, potentially improving the precision of SCPP assessment and guiding drainage strategies (104).

An EACTS position paper suggests maintaining MAP ≥ 90 mmHg and reducing CSFP to ≤10 mmHg to achieve an SCPP ≥ 80 mmHg although these targets are primarily supported by expert consensus rather than randomized evidence(Level IIaC evidence) (105). Consistently, the American Association of neurological surgeons and the congress of neurological surgeons (AANS/CNS) guidelines for acute spinal cord injury recommend maintaining MAP between 85 and 90 mmHg for the first 7 days after injury to optimize spinal cordo perfusion and potentially enhance neurological recovery (106).

In practice, while MAP ≥ 90 mmHg is widely adopted for prophylaxis and rescue of SCI, lower thresholds (around 65 mmHg) may occasionally be accepted in patients under full anticoagulation and extracorporeal support (e.g., cardiopulmonary bypass, left heart bypass), provided that SCPP is optimized, CSF drainage is available and neuromonitoring confirms cord integrity (107).

In such setting, individualized SCPP-guided titration becomes paramount, as MAP-SCPP relationship is non-linear and patient-specific. Measuring SCPP, however, is technically challenging, requiring intrathecal or intralesional pressure monitoring that is not widely available and may yield discrepant values depending on the monitoring site, particularly when the subarachnoid space is compromised by cord swelling or extradural compression. Consequently, current guidelines continue to rely on MAP-based recommendations as a pragmatic standard. Nevertheless, SCPP is increasingly recognized as a more precise surrogate of spinal cord perfusion than MAP alone and ongoing trial (e.g., CASPER, NCT03911492) are expected to provide critical evidence to refine future recommendations (108).

Experimental data support the protective role of elevated MAP during spinal cord ischemia. In a rabbit model of aortic cross-clamping, Izumi et al. (109) demonstrated that animals maintained at higher MAP (∼122 mmHg) exhibited increased spinal cord blood flow, reduced oxidative stress, accelerated recovery of motor evoked potentials and diminished histopathological damage. Similarly, in a rat model of ischemia- reperfusion, Lu et al. (110) showed that hypovolemia induced hypotension exacerbated neurological deficits and neuronal necrosis. Clinical experience underscores the benefit of maintaining SCPP >80 mmHg, particularly through MAP augmentation and CSF drainage, in both the prophylaxis and management of SCI. In cases of postoperative neurological deficit, a MAP target >90 mmHg should be pursued, with a gradual tapering of vasopressor support over 24–48 h following clinical improvement.

Evidence appraisal: Targets such as MAP ≥ 90 mmHg (and SCPP ≥80 mmHg) are mainly grounded in expert consensus and small observational cohorts, supported mechanistically by pre-clinical data (LoE C).

#### 5.2.3 Cerebrospinal fluid drainage: indications, timing and controversies

CSF is a widely employed adjunctive measure in both open and endovascular repair of thoracic and thoracoabdominal aortic aneurysm, owing to its ability to enhance spinal cord perfusion pressure (SCPP), directly determined by the difference between mean arterial pressure (MAP) and CSFP. During ischemic events, increased CSF production leads to elevated intraspinal pressure and consequent reduction in SCPP, thereby propagating spinal cord ischemic injury. By reducing CSFP, drainage effectively restores SCPP and is thus routinely used perioperatively in high-risk patients. The 2010 guidelines by the American Heart Association/American College of cardiology Foundation, subsequently reaffirmed by the 2022 guidelines, recommended CSF drainage during open and endovascular TAAA/TAA repair; however, the supporting evidence was limited to open procedures (111). In 2015, the European Association for Cardiothoracic Surgery (EACTS) extended this recommendation to thoracic endovascular aortic repair (TEVAR), endorsing CSF drainage in patients at high-risk for SCI, albeit based on level IIaC evidence (expert consensus). The Society also advised maintaining the drainage catheter for at least 48 h postoperatively to reduce the risk of delayed SCI onset. More recently, the 2024 EACTS/STS guidelines have reaffirmed the pivotal role of CSF drainage, strongly recommending its use in open TAAA replacement (Class I, level of evidence B). In addition, for patients at increased risk of SCI undergoing endovascular thoracic or thoracoabdominal repair, prophylactic CSF drainage should be also considered (Class IIa, level C) (112).

**Figure 4:**
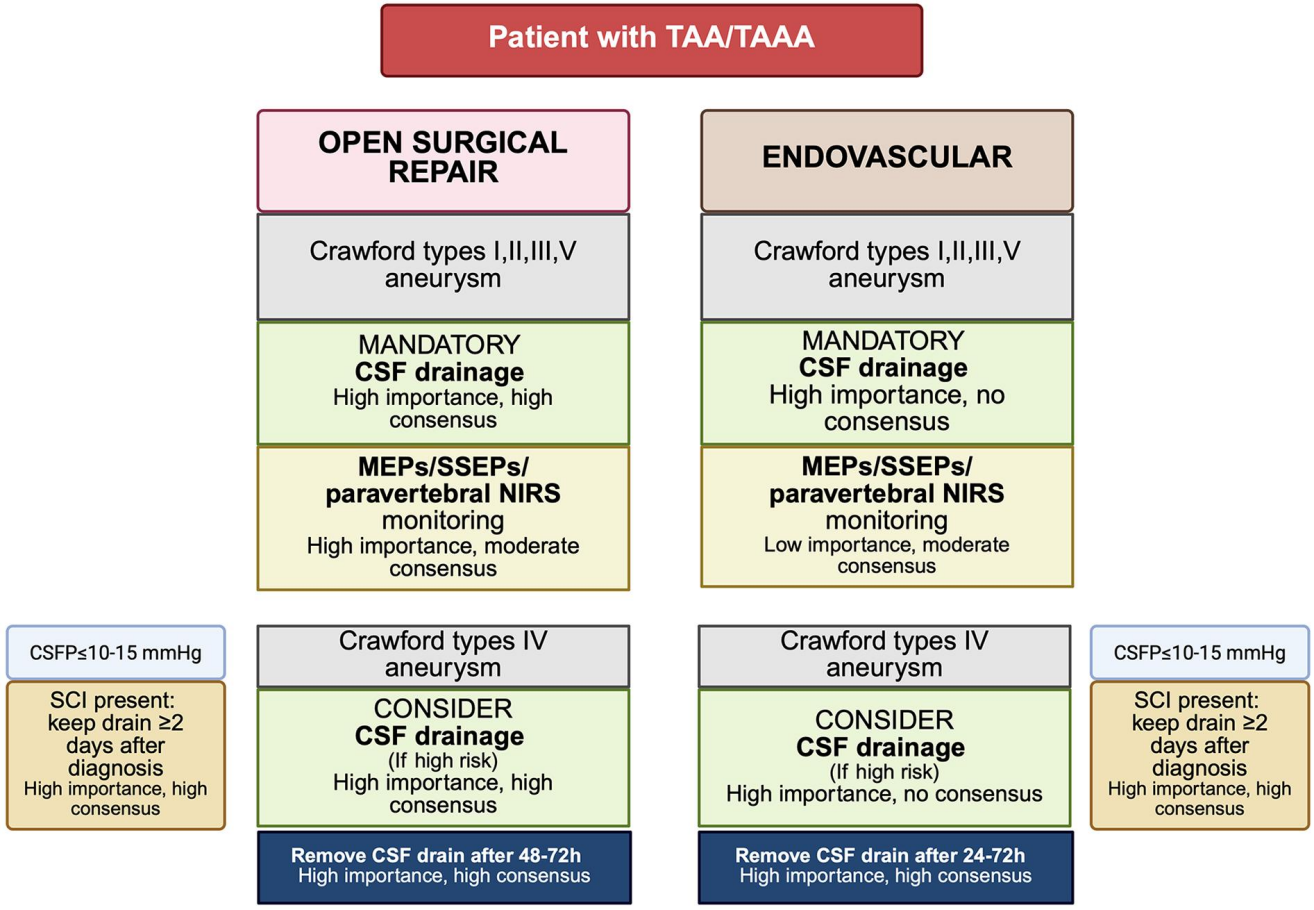
Proposed algorithm for CSF drain placement and neuromonitoring based on Delphi consensus recommendations (115). TAAA, thoracoabdominal aortic aneurysm; TAA, thoracic aortic aneurysm; CSF, cerebrospinal fluid; CSFP, cerebrospinal fluid pressure; MEPs, motor evoked potentials; SSEPs, somatosensory evoked potentials; NIRS, near-infrared spectroscopy; SCI, spinal cord injury. Created in BioRender. Pirri, C. (2025) https://BioRender.com/56whrg2, licensed under Academic License.

Risk stratification for SCI is variable across institutions, yet patients with Crawford type II or III aneurysm, a history of prior aortic surgery or occlusive disease involving the internal iliac or vertebral arteries are generally considered high risk (36). In these individuals, prophylactic CSF drainage is often adopted. Nevertheless, its routine preoperative placement remains controversial. Critics argue that the clinical benefit of prophylactic drainage has not been definitively demonstrated, particularly in the context of delayed SCI and that the risk of drainage related complications may outweigh potential benefits. Notably, Coselli et al. (113) demonstrated in a randomized controlled trial that CSF drainage significantly reduced the incidence of paraplegia/paraparesis in open TAAA repair (13% vs. 2.6%). However, comparable high-level evidence is lacking for TEVAR. A systematic review by Wong et al. (24) encompassing 4,936 patients found insufficient data to support clear role for prophylactic CSF drainage due to the absence of randomized trials. Similarly, Uchida's comprehensive review of endovascular thoracic aortic interventions from 1999 to 2013 yielded proposed, but unvalidated, indications for CSF drainage, emphasizing the need for prospective trials (114). Prophylactic drainage protocols remain heterogeneous (115) (Figure 4).

While some centers advocate catheter placement the day before surgery to allow early recognition of procedure-related complications, others favor intraoperative placement to reduce hospitalization time and cost (116). DeSart et al. (10) found no significant difference in long term functional outcomes based on timing, although early placement facilitated immediate intervention in cases of neurologic deterioration. Indeed, in patients developing postoperative SCI, the therapeutic window for effective reversal of neurologic deficits likely narrows to within 1–2 h, underscoring the utility of preemptive drainage. Routine drainage may also augment the efficacy of adjunctive therapies such as induced hypertension, which, in the absence of CSF decompression, may exacerbate spinal cord edema (117). Moreover, delayed drain placement is complicated by the development of post-stent coagulopathy, characterized by consumptive coagulopathy and activation of fibrinolytic pathways following aortic stent deployment (118). An alternative strategy, commonly referred as “wait and see” approach is feasible in TEVAR due to fast track anesthesia protocols enabling early neurologic assessment. However, successful implementation demands structured ICU monitoring, minimization of sedation and immediate access to trained personnel for urgent drain placement. Acher et al. (119), in a review of 22 studies, noted that SCI incidence remained between 1%–10%, irrespective of whether CSF drainage was administered routinely or selectively, or whether placement occurred intra or postoperatively. Despite its utility, CSF drainage is not without risks. Hanna et al. (42), reported an 11.1% rate of minor complications, including spinal headache, CSF leak and asymptomatic subdural hematoma, in 81 TEVAR patients. In contrast, Arnaoutakis et al. (116) observed no complications in a series of 48 patients, likely due to preoperative catheter placement and meticulous perioperative anticoagulation management. Antiplatelet agents, such as clopidogrel were withheld 7–10 days prior to drain insertion and low molecular weight heparin was discontinued 12 h before. Although the American Society of Regional anesthesia and Pain medicine guidelines on regional anesthesia in patients receiving antithrombotic therapy do not specifically address lumbar drain placement, their recommendation for neuraxial procedures have been widely extrapolated to this setting. Accordingly, maintaining a platelet count ≥ 100 × 10^3^/μl and an INR < 1.3 (with a normal aPTT) is generally regarded as the minimum requirement to minimize the risk of neuraxial bleeding (120). These thresholds, although not validated by dedicated randomized evidences, have been consistently endorsed in clinical practice. Notably Fedorow et al. (121) incorporated these criteria into best practice protocols for CSF drainage. This reliance on extrapolated thresholds underscores the paucity of dedicated evidence for spinal drain placement and highlights the need for procedure specific safety data. Excessive drainage rates (>15–20 ml/h) should also be avoided to mitigate the risk of clinically significant subdural hematoma. In practice, the majority of expert protocols aim to maintain CSFP below 8–15 mmHg, limiting drainage to a maximum of 15–20 ml/h. In asymptomatic patients, the drain is typically maintained for 24–72 h, depending on the estimated SCI risk. In the event of new onset neurologic symptom or neuromonitoring changes, the target CSFP may be further reduced <5 mmHg and subsequently titrated upward following neurologic recovery. The decision to remove or maintain the drain should always individualized according to the patient's clinical condition in the intensive care unit.

Evidence appraisal: In open TAAA, CSF drainage is supported by randomized evidence and guideline endorsement (Class 1 LoE B). For high-risk TEVAR evidence is observational and consensus-based (Class IIa, LoE C).

#### 5.2.4 Drugs and hypothermia

Neuroprotective strategies such as pharmacologic agents and hypothermia aim to reduce metabolic demand, limit apoptosis and inflammation and enhance ischemic tolerance. Glucocorticoids (methylprednisolone) show anti-inflammatory effects in animal models but lack clinical evidence for routine use and their risk must be weighed carefully (103, 122, 123). Intrathecal papaverine may improve perispinal blood flow and reduce SCI incidence in open repair, though data are limited (124). Other agents (naloxone, mannitol, barbiturates) have mechanistic or preclinical rationale but no robust clinical validation (125–127). Hypothermia (32–25°C) reduces cellular metabolism and has been applied in open aortic surgery (128). Cambria et al. (129) reported reduced SCI with epidural cooling using cold saline, but neither epidural nor systemic hypothermia has shown clear superiority. Systemic hypothermia, usually with cardiopulmonary bypass at ≈34°C, carries bleeding and resource burdens, requiring selective use (130).

Evidence appraisal: No drug therapy has robust outcome-level evidence for spinal cord protection in aortic surgery (consensus-based, LoE C). Systemic hypothermia for open TAAA is supported by low level evidence (LoE C).

#### 5.2.5 Distal aortic perfusion

Distal aortic perfusion (DAP) represents a cornerstone strategy in the protection of the spinal cord and distal organs during open thoracoabdominal aortic aneurysm repair. The rationale behind DAP lies in its ability to maintain perfusion below the site of aortic cross-clamping, thereby mitigating ischemic insult to the spinal cord, visceral organs and lower extremities (131). Unlike systemic or regional hypothermia, which solely reduced metabolic demand, DAP provides continuous delivery of oxygenated blood to ischemia-prone territories during aortic interruption. Technically, DAP is most commonly achieved through a left heart bypass circuit. This involves cannulation of the left atrium, typically via the left inferior pulmonary vein, to drain oxygenated blood. The blood is then pumped, using a centrifugal pump, through a heat exchanger and reinfused into the distal aorta, usually via femoral or iliac artery cannulation. The flow is non pulsatile and is regulated to achieve a distal aortic pressure of 60–80 mmHg, sufficient to maintain spinal cord and visceral perfusion. The protective effect of DAP is particularly crucial in Crawford type II repairs, where extensive aortic replacement involves cross-clamping above the celiac axis and includes interruption of multiple segmental arteries. In this context, DAP is often combined with mild systemic hypothermia and CSF drainage as part of a comprehensive multimodal approach aimed at preventing spinal cord ischemia. Safi et al. demonstrated in a large series of open TAAA repairs that the use of left heart bypass in conjunction with CSF drainage resulted in lower permanent paraplegia rate (132).

A Cochrane review likewise confirmed that adjuncts such as DAP are associated with a reduction in neurologic complications in high-risk TAAA procedures (133).

The European Association for cardio-thoracic surgery (EACTS), in a recent joint position paper on spinal cord protection, specifically recommend the use of distal perfusion techniques, most notably left heart bypass, as essential adjuncts during extensive open aortic procedures, particularly when the thoracic aorta is cross-clamped for prolonged periods. While DAP is highly effective, it requires significant technical expertise and specialized equipment and carries inherent risks, including atheroembolism, bleeding at the cannulation sites and complications related to anticoagulation (112).

Beyond distal aortic protection, left-heart bypass, CBP can be configured to maintain hepato-splanchnic perfusion during extensive open repairs, supporting visceral oxygen delivery while the descending aorta is interrupted. This strategy requires a dedicated perfusion team and meticulous anticoagulation management (113, 134).

Evidence appraisal: Early restoration of distal and visceral/renal perfusion is supported by prospective cohorts and systematic reviews (LoE B), with guideline recommendation favoring selective perfusion strategies in complex open repair.

### 5.3 Therapeutic strategies for spinal cord injury in TAA/TAAA surgery

#### 5.3.1 Intraoperative SCI onset

During TAA/TAAA repair, vigilant real-time interpretation of intraoperative neuromonitoring (IONM) data is essential for early detection of spinal cord ischemia and for guiding prompt corrective measures aimed at restoring adequate spinal cord perfusion. A decline in MEPs or SSEPs should immediately prompt strategies to augment mean arterial pressure (MAP) above 90 mmHg, typically through judicious fluid resuscitation and vasopressor administration. In high-risk patients with preoperatively placed lumbar drains, CSFP should be maintained below 10 mmHg through active drainage to optimize SCPP. In patients who develop intraoperative SCI without a previously placed lumbar drain, a therapeutic drain may be inserted at the conclusion of the procedure. According to recommendations by Aucoin et al. on behalf of the US Aortic Research Consortium, rescue strategies should also include maintaining hemoglobin concentrations ≥10 g/dl, ensuring MAP remains ≥90 mmHg and obtaining immediate postoperative spinal imaging to exclude compressive hematomas (135). Although high-dose corticosteroids have been employed in conjunction with these interventions, current evidences do not support a uniform recommendation for their routine use.

#### 5.3.2 Postoperative SCI onset

Meticulous hemodynamic and neurologic surveillance throughout the postoperative course is paramount in patients undergoing TAA/TAAA repair. A low threshold for initiating rescue strategies is essential to minimize the risk of irreversible SCI and to promote neurologic recovery in patients exhibiting early signs of dysfunction. NIRS may be employed in the intensive care unit to monitor high-risk patients, although current evidence does not support a standardized cutoff value for SCI detection. In patients developing postoperative neurologic deficits, the same principles applied intraoperatively should be promptly adopted. Hemodynamic goals include maintaining a MAP above 90 mmHg and a hemoglobin concentration at least 10 g/dl to optimize spinal cord oxygen delivery. If a prophylactic CSF drain was not placed preoperatively, therapeutic drainage should be initiated with the aim of keeping CSFP below 10 mmHg. Therapeutic CSF drainage may also be beneficial in patients who develop delayed onset SCI, with several reports documenting favorable neurologic outcomes (136). Furthermore, permissive hypertension, maintaining systolic blood pressure up to 150 mmHg, has been suggested as a protective strategy in early postoperative phase, particularly within the first month (137). Lastly, although high-dose corticosteroids have been incorporated into some rescue protocols, including those for delayed SCI, their efficacy remains unproven and current guidelines do not endorse their routine use (138).

A novel regenerative therapeutic approach currently under investigation is shock wave therapy (SWT), which has shown promise in attenuating oxidative stress and promoting neuronal survival after spinal cord ischemia. Preliminary human data suggest that SWT may enhance functional recovery when applied after the onset of neurologic injury, possibly through activation of the TLR3-NRF2 signaling axis (139, 140). While early results are encouraging, further validation in larger, controlled clinical trials is warranted before its incorporation into standard rescue protocols.

#### 5.3.3 Early recognition and rehabilitation

From a neurorehabilitation perspective, early recognition of peri-and postoperative SCI- is an absolute requirement. Through neurological examination and careful differential diagnosis should be systematically performed, as incomplete SCI may remain undetected or be misattributed to conditions such as critical illness polyneuropathy or decompensation of preexisting comorbidities, particularly in frail patients. Prompt awareness is crucial, since timely detection allows early referral to specialized spinal cord injury rehabilitation centers, where state-of-the art, multidisciplinary care can significantly influence long-term functional recovery. Access to dedicated SCI rehabilitation should be consider an integral part of comprehensive perioperative management in aortic surgery (141, 142).

## 6 Discussion

Spinal cord injury remains one of the most feared complications of TAA and TAAA repair, both open surgical and endovascular approaches. Despite significant advancements in surgical techniques, perioperative management and device design, its incidence remains substantial, reflecting the complexity of spinal cord perfusion and the vulnerability of this tissue to ischemia. The pathophysiology is multifactorial, involving perfusion pressure, collateral network integrity and embolic burden from surgical manipulation or endovascular deployment (143). While the artery of Adamkiewicz has historically been regarded as crucial, recent evidence highlights the dominant role of the collateral network, including intraspinous and paraspinous pathways, in maintaining viability when segmental arteries are excluded (144).

Open repair, especially in extent I-III aneurysm, entails profound hemodynamic perturbations such as elevated central venous and intracranial pressures combined with distal hypoperfusion, markedly reducing spinal cord perfusion pressure (135). Ischemia-reperfusion after aortic unclamping further exacerbates damage. TEVAR, while avoiding some of these derangements, carries a distinct risk through extensive segmental artery coverage and progressive remodeling of the spinal vascular network (145). Early paraplegia may occur with abrupt hypoperfusion when collateral reserve is insufficient, whereas delayed deficits often reflect evolving ischemia in watershed regions, unmasked by systemic hypotension, arrhythmias or thromboembolic events. Neuroprotective strategies aim to preserve SCPP and mitigate both early and delayed deficits. CSF drainage remains a cornerstone, endorsed by American Heart Association (AHA) and international consensus (11, 111).

By lowering intrathecal pressure and coupled with MAP augmentation, CSF drainage improves SCPP and offers therapeutic benefit even in delayed SCI, which can be partially reversible in up to 57% of patients (11). Current guidelines recommend prophylactic CSF drainage in open or endovascular repair of Crawford extent I, II, III and V aneurysms and in extent IV repairs when additional risk factors are present (115, 145). Standardized protocols now provide guidance on target pressures (≤10–15 mmHg), timing and safe removal within 24–72 h depending on repair type and neurological status (115, 145, 146). Another key consideration in TEVAR is LSA coverage, required in up to 40% of cases. Without revascularization, this maneuver increases the risk of vertebrobasilar insufficiency, stroke and SCI. Preoperative LSA revascularization is strongly recommended in selected high-risk scenarios and reduces both cerebrovascular and spinal complications. Emerging strategies such as staged repair and MISACE aim to precondition the collateral network, enhancing spinal cord resilience to ischemia (85). Similarly, intentional endoleaks have been proposed to sustain perfusion during the perioperative window, although further evidence is required (87). Recent studies have also explored biomarkers for early SCI detection, including GFAP, NFL, tau protein, S100B and NSE, which reflect astroglial damage, axonal injury and blood-spinal cord barrier disruption (147–150). While these rise within hours and correlate with tissue injury, clinical validation remains incomplete with unresolved questions about sampling, timing and thresholds. Future models will likely integrate such biomarkers with physiological metrics, monitoring data and procedural variables to improve early detection and rescue strategies. Looking ahead, a precision medicine approach that integrates patient-specific risk stratification, real-time perfusion monitoring and adaptive neuroprotective strategies is anticipated to define the next frontier. Advanced imaging, such as perfusion MRI and 4D flow MRI, may offer direct visualization of spinal vascular territories, while multicenter registries and randomized trials are urgently needed to validate protection bundles, biomarkers and innovative approaches like MISACE and staged repair.

### 6.1 Conclusions

Spinal cord protection in thoracic and thoracoabdominal aortic surgery relies on anticipatory risk stratification and consistent implementation of bundle-based care that integrates neuromonitoring, protocolized CSF management, pragmatic hemodynamic goals and center-specific perfusion strategies. In open repair, distal perfusion and selective use of hypothermia with cardiopulmonary bypass, remain pivotal, while in endovascular repair the priorities include minimizing unnecessary coverage, managing the LSA and structured postoperative surveillance. Because SCI may occur in delayed fashion, institutions must maintain explicit rescue pathways involving MAP optimization, CSF drainage, hemoglobin and oxygen delivery management, urgent imaging and revascularization when indicated. Research priorities include prospective evaluation of standardized bundles, development of multimodal predictive models, combining physiological, imaging and biomarker data and validation of non-invasive monitoring technologies. Advancing these strategies will be crucial to move from consensus-driven practice toward robust, evidence-based, personalized protection across techniques and centers.
